# Age‐related DNA methylation on Y chromosome and their associations with total mortality among Chinese males

**DOI:** 10.1111/acel.13563

**Published:** 2022-02-04

**Authors:** Guyanan Li, Chenming Wang, Xin Guan, Yansen Bai, Yue Feng, Wei Wei, Hua Meng, Ming Fu, Meian He, Xiaomin Zhang, Yanjun Lu, Yong Lin, Huan Guo

**Affiliations:** ^1^ Department of Occupational and Environmental Health State Key Laboratory of Environmental Health (Incubating) School of Public Health Tongji Medical College Huazhong University of Science and Technology Wuhan China; ^2^ Department of Clinical Laboratory Medicine Shanghai Fifth People's Hospital Fudan University Shanghai China; ^3^ Department of Laboratory Medicine Tongji Hospital Tongji Medical College Huazhong University of Science and Technology Wuhan China; ^4^ Department of Laboratory Medicine Huashan Hospital Fudan University Shanghai China; ^5^ National Clinical Research Center for Aging and Medicine Huashan Hospital Fudan University Shanghai China

**Keywords:** aging, DNA methylation, mortality, Y chromosome

## Abstract

In view of the sex differences in aging‐related diseases, sex chromosomes may play a critical role during aging process. This study aimed to identify age‐related DNA methylation changes on Y chromosome (ChrY). A two‐stage study design was conducted in this study. The discovery stage contained 419 Chinese males, including 205 from the Wuhan‐Zhuhai cohort panel, 107 from the coke oven workers panel, and 107 from the Shiyan panel. The validation stage contained 587 Chinese males from the Dongfeng‐Tongji sub‐cohort. We used the Illumina HumanMethylation BeadChip to determine genome‐wide DNA methylation in peripheral blood of the study participants. The associations between age and methylation levels of ChrY CpGs were investigated by using linear regression models with adjustment for potential confounders. Further, associations of age‐related ChrY CpGs with all‐cause mortality were tested in the validation stage. We identified the significant associations of 41 ChrY CpGs with age at false discovery rate (FDR) <0.05 in the discovery stage, and 18 of them were validated in the validation stage (*p* < 0.05). Meta‐analysis of both stages confirmed the robust positive associations of 14 CpGs and negative associations of 4 CpGs with age (FDR<0.05). Among them, cg03441493 and cg17816615 were significantly associated with all‐cause mortality risk [HR(95% CI) = 1.37 (1.04, 1.79) and 0.70 (0.54, 0.93), respectively]. Our results highlighted the importance of ChrY CpGs on male aging.

## Graphical abstract

The methylation levels of 18 CpGs on Y chromosome in peripheral blood were found to be significantly associated with age. The methylation levels of *TTTY14* and *EIF1AY* were negatively associated with their gene expression levels. The methylation levels of cg03441493 and cg17816615 were significantly associated with all‐cause mortality risk among Chinese males.

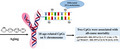

**Abbreviations**
ChrY: Y chromosome
COW: coke oven workers
CpG: cytosine‐phosphate‐guanine dinucleotide
*DDX3Y*
: *DEAD*‐*box helicase 3 Y*‐*linked*
DFTJ cohort: Dongfeng‐Tongji cohort
*EIF1AY*
: *eukaryotic translation initiation factor 1A Y*‐*linked*
FDR: false discovery rate
INT: inverse normal transformed
MRS: methylation risk score
*NLGN4Y*
: *neuroligin 4 Y*‐*linked*
SY: Shiyan
*TBL1Y*
: *transducin beta like 1 Y*‐*linked*
*TMSB4Y*
: *thymosin beta 4 Y*‐*linked*
*TTTY13*
: *testis*‐*specific transcript Y*‐*linked 13*
*TTTY14*
: *testis*‐*specific transcript Y*‐*linked 14*
*TTTY15*
: *testis*‐*specific transcript Y*‐*linked 15*
WHZH cohort: Wuhan‐Zhuhai cohort

## 1 INTRODUCTION

Aging is a slow, time‐dependent decline of biological functions accompanied by the impaired ability to respond to stress, increased homeostatic instability, and an increased risk of related diseases (Dorronsoro et al., 2021). According to the seventh national census in 2020, the proportion of elderly people aged 60 and above in China is around 18.7%. The key to address increasingly serious aging issues is to obtain the reliable aging biomarkers, which has enormous significance in explaining the mechanisms of aging and predicting the future health outcomes.

Aging is influenced by both genetic and environmental factors (de Magalhaes et al., 2012). Genetics seems to explain a small fraction of the variation in the human life span, while the role of epigenetics is also essential in aging process (Brooks‐Wilson, 2013). Recently, a growing body of evidence has found that DNA methylation, which occurs in the context of a cytosine‐phosphate‐guanine dinucleotide (CpG), varies substantially with age over a long time period (Jones et al., 2015). Weighted sums of methylation at multiple age‐related CpG sites are considered as “epigenetic clocks,” which are highly correlated with chronological age and may predict age‐related health outcomes (Gibson et al., 2019). In 2013, Horvath et al proposed a pan‐tissue epigenetic clock based on the methylation pattern of 353 CpGs (Horvath, 2013). Then, Hannum constructed an epigenetic age clock derived from the linear combination of methylation levels at 71 CpGs (Hannum et al., 2013). Following these pioneering works, Levine et al identified a set of 513 CpGs that could predict “PhenoAge,” which was informed by chronological age and physical as well as biochemical measures (Levine et al., 2018). However, these currently reported age‐related CpGs are mainly located on the autosomal chromosomes, while age‐dependent changes in DNA methylation on sex chromosomes have typically been ignored.

The gender disparity of aging has been widely reported. On an average, men's life expectancy is shorter than that of women around the world (66.4 years old vs. 70.4 years old) (Forsberg, 2017). In addition, men generally have higher incidence and poorer survival for most aging‐related diseases (Radkiewicz et al., 2017). On the epigenetic aspect of aging, men also exhibit increased acceleration in DNA methylation age than women, suggesting that the epigenetic changes in sex chromosomes may play an important role in determining sex‐specific longevity (McCartney et al., 2019). Y chromosome (ChrY) is a male‐specific sex chromosome, which contains 57 million DNA base pairs and harbors 78 protein‐coding genes (Bachtrog, 2013; Lund et al., 2020). It has been reported that DNA methylation pattern on human ChrY is evolutionarily conserved and remains stable among family members (Zhang et al., 2016). Many previous literatures about ChrY and aging have mainly focused on the mosaic loss of ChrY, but few studies have pointed out the importance of ChrY DNA methylation pattern on aging (Forsberg, 2017; Guo et al., 2020; Thompson et al., 2019). Lund et al identified the significant associations of seven Y‐CpGs with age among 624 elderly men from four European cohorts (56.99–94.97 years old, mean age: 77.66 years old) (Lund et al., 2020). Recently, Vidaki et al used DNA methylation microarray data of 1,057 European males aged 15–87 years old from six publicly available Gene Expression Omnibus datasets and found 19 age‐dependent ChrY CpGs by using machine learning algorithms (Vidaki et al., 2021). However, due to the marked racial and ethnic disparities in aging, further investigations about aging and DNA methylation on ChrY among other ethnic males are also needed (Weuve et al., 2018).

In the present study, we performed an epigenome‐wide analysis of age with DNA methylation on ChrY by using a two‐stage study design among Chinese males. For the significant age‐related CpG sites on ChrY, we further evaluated their associations with all‐cause mortality among the males in the Dongfeng‐Tongji (DFTJ) sub‐cohort.

## 2 RESULTS

### 2.1 Basic characteristics of study participants

There were 419 and 587 males in the discovery stage and validation stage, respectively. The general characteristics of study participants were presented in Table 1. In the discovery stage, the mean (min–max) age for male participants in the Wuhan‐Zhuhai (WHZH) cohort panel, the Shiyan (SY) panel, and the coke oven workers (COW) panel were 53.3 (25.6–89.0), 40.0 (22.0–65.0), and 47.0 (26.2–60.2) years old, respectively. The mean (min–max) age for male subjects in the validation stage of DFTJ sub‐cohort was 66.6 (42.5–87.5) years old.

**TABLE 1:** General characteristics of study participants

Variables	Discovery stage			Validation stage
	WHZH panel	SY panel	COW panel	DFTJ sub‐cohort
No. (males)	205	107	107	587
Age (years)				
Mean ± SD	53.3 ± 12.6	40.0 ± 10.7	47.0 ± 9.4	66.6 ± 6.6
min–max	25.6–89.0	22.0–65.0	26.2–60.2	42.5–87.5
BMI (kg/m^2^)	23.6 ± 2.8	24.5 ± 2.6	23.8 ± 2.7	24.0 ± 3.0
Smoking pack‐year	14.6 ± 14.9	8.3 ± 14.0	20.1 ± 17.5	18.0 ± 20.9
Smoking status				
Ever smokers	133 (64.9)	47 (43.9)	87 (81.3)	353 (60.1)
Never smokers	72 (35.1)	60 (56.1)	20 (18.7)	234 (39.9)
Alcohol drinking status				
Ever alcohol drinkers	83 (40.5)	54 (50.5)	51 (47.7)	318 (54.2)
Never alcohol drinkers	122 (59.5)	53 (49.5)	56 (52.3)	269 (45.8)
White blood cell counts (×10^9^/L)				
Total white blood cells	6.1 ± 1.6	6.1 ± 1.4	6.9 ± 1.6	5.6 ± 1.3
Neutrophils	3.4 ± 1.1	3.5 ± 1.0	4.2 ± 1.3	3.4 ± 1.0
Lymphocytes	2.2 ± 0.7	2.1 ± 0.6	2.5 ± 0.6	1.7 ± 0.5
Intermediate cells	0.4 ± 0.3	0.5 ± 0.2	0.3 ± 0.1	0.5 ± 0.2

Note

Values were shown as mean ± SD for continuous variables or *n* (%) for categorical variables. Intermediate cells were the sum of monocytes, eosinophils, and basophils.

Abbreviations: BMI, body mass index; COW, study subjects selected from the cohort of coke oven workers; DFTJ, sub‐cohort subjects from the Dongfeng‐Tongji Cohort Study; SY, subjects recruited from Shiyan City, Hubei Province, China; WHZH, study subjects selected from the Wuhan‐Zhuhai cohort study.

### 2.2 Age‐related changes in DNA methylation on ChrY

We found the greater variations in methylation levels of ChrY CpGs compared with those on the autosomal and ChrX counterparts (*p* < 0.001, Figure S1). The distribution of *β*‐values of ChrY CpGs in each study panel showed a consistent bimodal distribution, with two peaks at *β* < 0.1 and *β* > 0.8 (Figure S2).

In the discovery stage, methylation levels of 41 ChrY CpGs showed significant associations with age after multiple testing correction [false discovery rate (FDR) <0.05, Table S1]. Of them, 36 CpGs (87.8%) were positively associated with age and 5 CpGs, including cg17816615 in *DEAD*‐*box helicase 3 Y*‐*linked* (*DDX3Y*), cg01988452, cg13308744, and cg10172760 in *eukaryotic translation initiation factor 1A Y*‐*linked* (*EIF1AY*), and cg14467015 in *testis*‐*specific transcript Y*‐*linked 13* (*TTTY13*) were negatively associated with age. Locations of age‐related hypermethylated and hypomethylated CpGs across the ChrY were presented in Figure S3.

The associations of age and above 41 CpGs were then tested in the validation stage. Among them, the methylation levels of 18 CpGs were significantly associated with age (*p* < 0.05, Table S1). Meta‐analysis of both stages confirmed the positive associations of age with the methylation levels of 14 CpGs [annotated to *transducin beta like 1 Y*‐*linked* (*TBL1Y*), *testis*‐*specific transcript Y*‐*linked 15* (*TTTY15*), *thymosin beta 4 Y*‐*linked* (*TMSB4Y*), *neuroligin 4 Y*‐*linked* (*NLGN4Y*), and *testis*‐*specific transcript Y*‐*linked 14 (TTTY14)*, respectively), as well as the significant negative associations of age with the methylation levels of 4 CpGs, cg17816615, cg01988452, cg13308744, and cg14467015, annotated to *DDX3Y*, *EIF1AY*, and *TTTY13*, respectively (Table 2).

**TABLE 2:** Associations between methylation levels of 18 CpGs on ChrY and age among study participants

CpG	Position	Mapped Gene	Relation to Gene	Discovery stage			Validation stage			Meta‐analysis of both stages			
				*β*	SE	*p*	*β*	SE	*p*	*β*	SE	*p*	FDR
cg01707559	6778695	*TBL1Y*	TSS200	3.51	0.66	1.95E−07	0.67	0.27	1.38E−02	1.08	0.25	1.80E−05	**3.24E−05**
cg03441493	14074417			2.08	0.56	2.63E−04	0.55	0.27	3.82E−02	0.83	0.24	5.74E−04	**6.88E−04**
cg09093035	14074689			3.09	0.55	4.18E−08	0.94	0.26	3.80E−04	1.34	0.24	1.97E−08	**1.18E−07**
cg03258315	14077975			2.31	0.69	9.58E−04	0.62	0.27	2.03E−02	0.84	0.25	7.88E−04	**8.34E−04**
cg09730640	14533708			3.36	0.56	3.33E−09	0.86	0.28	2.63E−03	1.38	0.25	5.06E−08	**1.82E−07**
cg25032547	14773536	*TTTY15*	TSS1500	2.14	0.58	2.23E−04	0.55	0.27	4.61E−02	0.84	0.25	6.34E−04	**7.13E−04**
cg17816615	15019332	*DDX3Y*	Body	−2.09	0.59	4.09E−04	−0.95	0.28	6.05E−04	−1.16	0.25	3.53E−06	**7.06E−06**
cg26198148	15814685	*TMSB4Y*	TSS1500	2.28	0.61	2.02E−04	0.95	0.28	6.20E−04	1.18	0.25	2.96E−06	**6.66E−06**
cg04691144	16634373	*NLGN4Y*	TSS1500	4.62	0.65	6.74E−12	0.70	0.30	1.75E−02	1.36	0.27	4.00E−07	**1.20E−06**
cg27443332	16634795	*NLGN4Y*	Body	2.52	0.57	1.03E−05	1.11	0.28	1.05E−04	1.40	0.25	4.08E−08	**1.82E−07**
cg03706273	16635745	*NLGN4Y*	1stExon	3.41	0.67	5.82E−07	0.61	0.29	3.36E−02	1.04	0.26	7.76E−05	**1.16E−04**
cg03244189	21238472	*TTTY14*	Body	2.06	0.63	1.11E−03	0.67	0.28	1.79E−02	0.90	0.26	4.43E−04	**5.69E−04**
cg13845521	21238886	*TTTY14*	Body	2.89	0.55	3.03E−07	0.78	0.28	6.09E−03	1.22	0.25	1.43E−06	**3.68E−06**
cg11816202	21239332	*TTTY14*	TSS200	2.56	0.57	8.43E−06	0.68	0.28	1.70E−02	1.05	0.25	3.16E−05	**5.17E−05**
cg15345074	21239461	*TTTY14*	TSS200	1.67	0.58	4.37E−03	0.59	0.27	3.12E−02	0.78	0.25	1.53E−03	**1.53E−03**
cg01988452	22736528	*EIF1AY*	TSS1500	−2.68	0.55	1.71E−06	−1.36	0.26	3.72E−07	−1.61	0.24	1.67E−11	**1.50E−10**
cg13308744	22736584	*EIF1AY*	TSS1500	−4.20	0.53	1.48E−14	−0.97	0.27	3.25E−04	−1.64	0.24	7.71E−12	**1.39E−10**
cg14467015	23757241	TTTY13	TSS1500	−2.07	0.63	1.11E−03	−0.72	0.26	6.88E−03	−0.92	0.24	1.53E−04	**2.12E−04**

Note

Association analyses were performed by using linear regression models in each stage, with inverse normal transformed DNA methylation *β* value as the independent variable, age as the dependent variable, with adjustment for BMI, smoking status, drinking status, white blood cell counts and experimental batch (only in the discovery stage). Results from discovery and validation stages were combined by using a fixed‐effect meta‐analysis.

**FIGURE 1:**
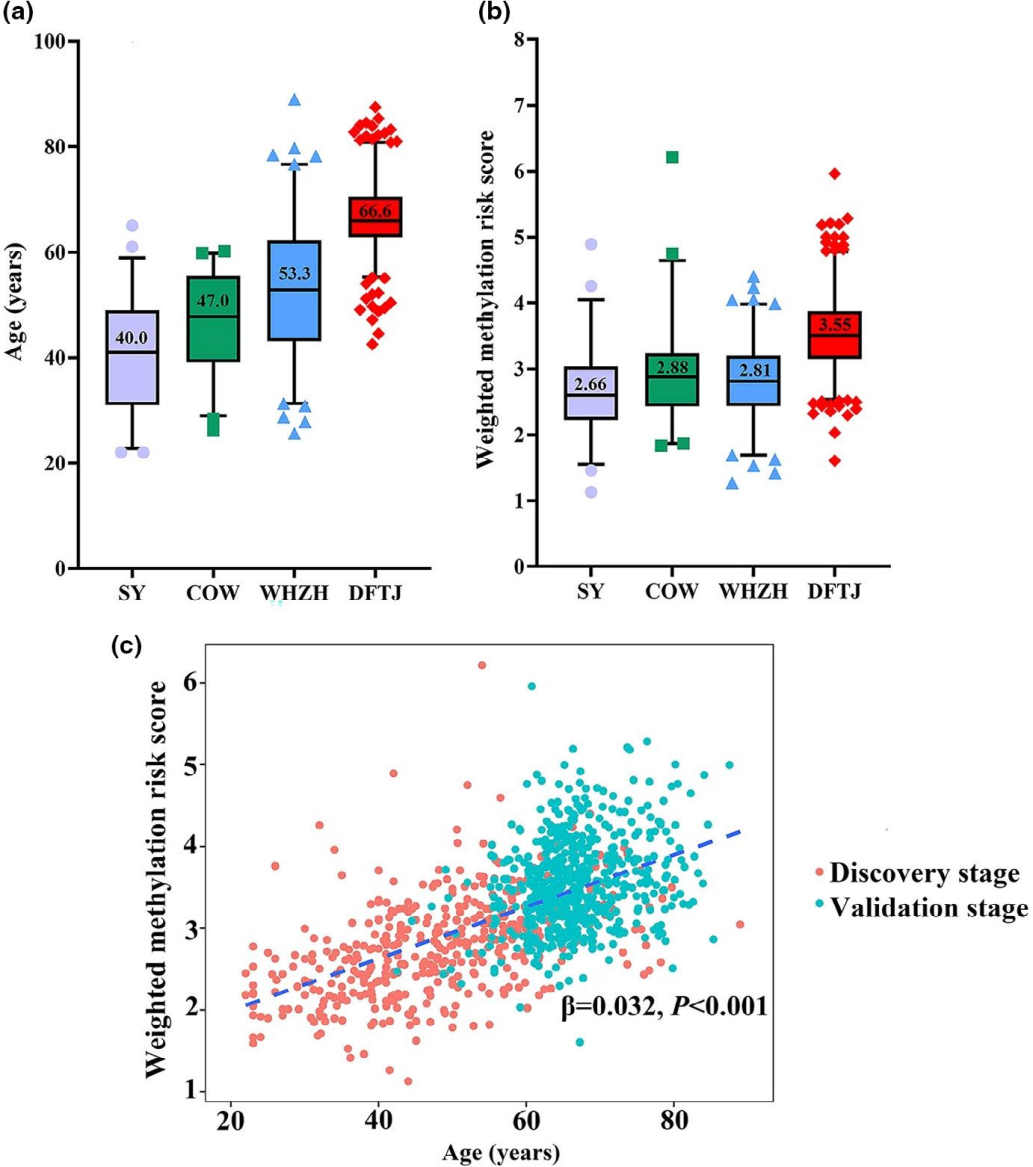
Weighted methylation risk score (MRS) on Y chromosome (ChrY) and age among participants. (a) Distribution of age in each study panel. (b) Distribution of weighted MRS on ChrY in each study panel. (c) Association of weighted MRS on ChrY with age. Trend line was fitted by linear regression model. Weighted MRS was calculated as the sum of their coefficients with age multiplied by methylation levels of corresponding CpGs

To exclude the effects of smoking and alcohol drinking on the associations between ChrY DNA methylation and age, we firstly tested the relationships of DNA methylation and smoking pack‐year/alcohol drinking status. We observed that only the methylation level of cg13845521 among the 18 CpGs showed suggestive significant association with smoking pack‐year [for each additional 10‐pack‐year, *β* (se) = 0.04 (0.02), *p* = 2.67E−02, Table S2], and the methylation levels of cg13845521 and cg11816202 in ever alcohol drinkers were lower than those in the never alcohol drinkers [*β* (se) = −0.19 (0.06), *p* = 2.20E−03 and *β* (se) = −0.15 (0.06), *p* = 1.24E−02, respectively] (Table S2). However, these differences did not reach the multiple comparison threshold. The further stratification analyses showed that the directions of associations were consistent in each stratum when stratified by smoking status (Table S3) and alcohol drinking status (Table S4). No significant modification effects of smoking status and alcohol drinking status were identified at Bonferroni corrected threshold (all *p*_interaction_ > 2.78E−03, 0.05/18 CpGs). Among participants from four panels in both stages, the mean age ranged from 40.0 to 66.6 years old. We also calculated the weighted methylation risk score (MRS) according to the weighted sums of the methylation values of age‐related ChrY CpGs and found a significant increased trend of weighted MRS with age (*β* = 0.032, Figure 1). The positive association of weighted MRS with age was also shown in subjects of each panel (all *p* < 0.001, Figure S4).

### 2.3 Correlations of DNA methylation on ChrY with gene expression levels

**FIGURE 2:**
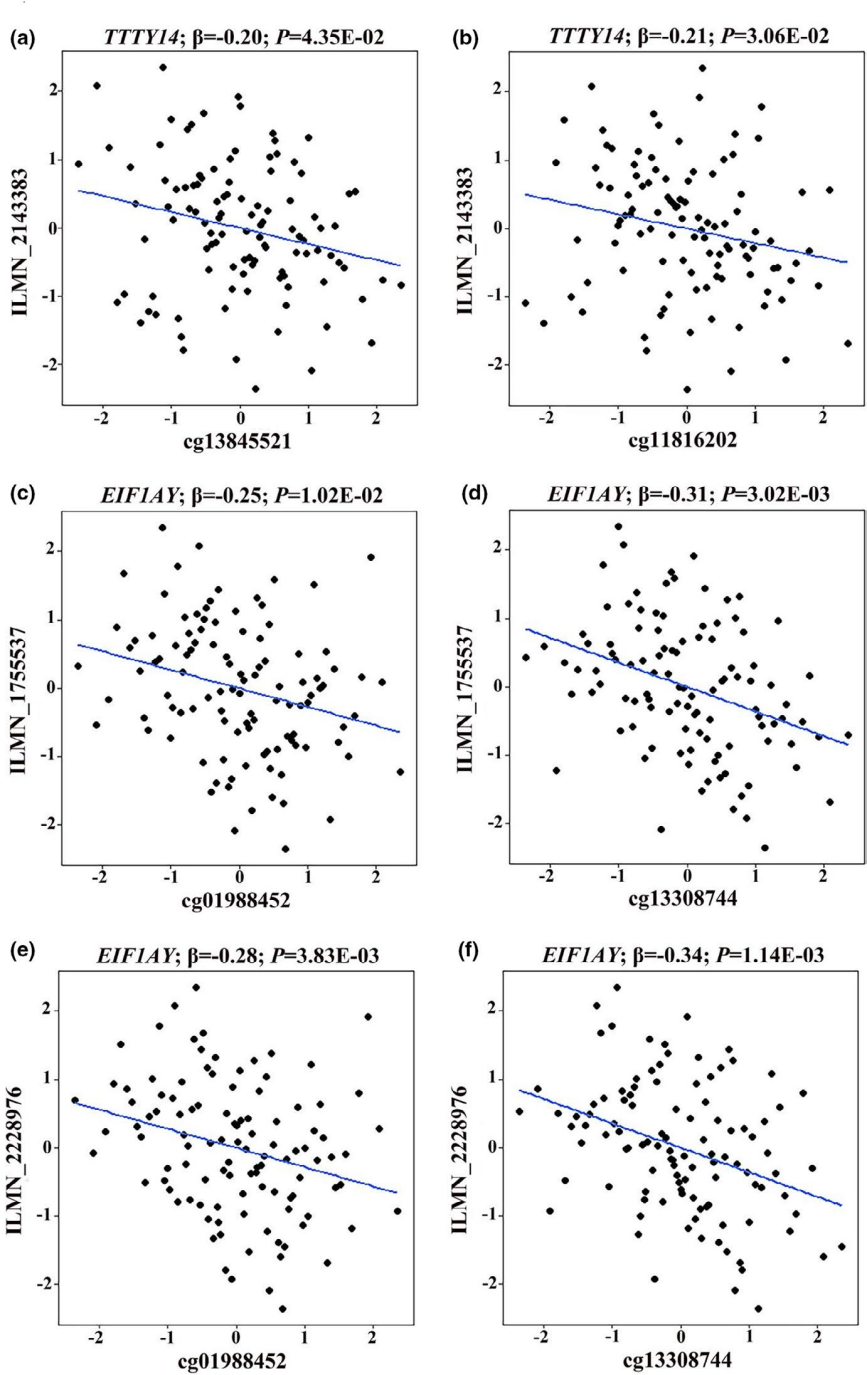
Methylation‐expression correlations for age‐related CpGs on ChrY. (a) and (b) correlations of cg13845521 and cg11816202 with the expression level of *TTTY14* (probe ILMN_2143383); (c) and (d) correlations of cg01988452 and cg13308744 with the expression level of *EIF1AY* (probe ILMN_1755537); (e) and (f) correlations of cg01988452 and cg13308744 with the expression level of *EIF1AY* (probe ILMN_2228976). Only methylation‐expression correlations with *p* < 0.05 were presented

To investigate whether the methylation of age‐related CpGs were correlated with the expression levels of their corresponding genes, we further detected the gene expression profiles in peripheral blood of males in the SY panel. It was shown that the methylation levels at cg13845521 and cg11816202 were negatively associated with the expression level of *TTTY14* (*β* = −0.20, *p* = 4.35E−02 and *β* = −0.21, *p* = 3.06E−02 for the associations of cg13845521 and cg11816202 with ILMN_2143383, respectively, Figure 2). Methylation levels at cg01988452 and cg13308744 were negatively associated with the expression levels of *EIF1AY* transcripts (*β* = −0.25, *p* = 1.02E−02 and *β* = −0.31, *p* = 3.02E−03 for the association with ILMN_1755537, respectively; *β* = −0.28, *p* = 3.83E−03 and *β* = −0.34, *p* = 1.14E−03 for the association with ILMN_2228976, respectively). While the methylation values of the other age‐related Y‐CpGs were not associated with the expression levels of their annotated genes (Table S5). In addition, Figure S5 showed the correlations between age and the expression levels of *TTTY14* (*β* = −0.02, *p* = 1.32E−02) and *EIF1AY* (*β* = 0.01, *p* = 1.26E−01 for ILMN_1755537; *β* = 0.01, *p* = 5.68E−02 for ILMN_2228976).

### 2.4 Associations between age‐related CpGs on ChrY and mortality risk among males in the DFTJ sub‐cohort

The above‐mentioned 587 males in the DFTJ sub‐cohort were all successfully followed up from 2013 to the end of 2018, and 55 (9.4%) among them died during this period. Among the 18 validated age‐associated CpGs on ChrY, the methylation level of cg03441493 showed a positive association with increased risk of all‐cause mortality [HR (95% CI) = 1.37 (1.04, 1.79), *p* = 2.48E−02], while the methylation level of cg17816615 in *DDX3Y* was negatively associated with all‐cause mortality [HR (95% CI) = 0.70 (0.54, 0.93), *p* = 1.25E−02] (Table 3). However, we did not find significant effects of the other ChrY CpGs and MRS on all‐cause mortality risk (all *p* > 0.05) in this population.

**TABLE 3:** Associations of age‐related DNA methylation on ChrY with all‐cause mortality in the validation stage

CpG	Position	Mapped Gene	Relation to Gene	Model 1			Model 2		
				HR	95% CI	*p*	HR	95% CI	*p*
cg01707559	6778695	*TBL1Y*	TSS200	1.06	(0.81, 1.40)	6.80E−01	1.08	(0.82, 1.43)	5.75E−01
cg03441493	14074417			**1.35**	**(1.05, 1.74)**	**2.65E−02**	**1.37**	**(1.04, 1.79)**	**2.48E−02**
cg09093035	14074689			0.97	(0.75, 1.25)	8.11E−01	0.97	(0.74, 1.28)	8.33E−01
cg03258315	14077975			1.21	(0.92, 1.59)	1.63E−01	1.20	(0.93, 1.54)	4.90E−01
cg09730640	14533708			0.99	(0.76, 1.31)	9.80E−01	0.95	(0.71, 1.28)	7.56E−01
cg25032547	14773536	*TTTY15*	TSS1500	0.86	(0.67, 1.11)	2.58E−01	0.84	(0.65, 1.09)	1.93E−01
cg17816615	15019332	*DDX3Y*	Body	**0.70**	**(0.54, 0.93)**	**1.21E−02**	**0.70**	**(0.54, 0.93)**	**1.25E−02**
cg26198148	15814685	*TMSB4Y*	TSS1500	0.94	(0.73, 1.22)	6.25E−01	0.92	(0.72, 1.19)	5.44E−01
cg04691144	16634373	*NLGN4Y*	TSS1500	0.96	(0.75, 1.24)	7.90E−01	0.99	(0.77, 1.27)	9.31E−01
cg27443332	16634795	*NLGN4Y*	Body	0.81	(0.62, 1.08)	1.50E−01	0.83	(0.63, 1.10)	1.83E−01
cg03706273	16635745	*NLGN4Y*	1stExon	0.97	(0.74, 1.28)	8.25E−01	0.97	(0.74, 1.28)	8.30E−01
cg03244189	21238472	*TTTY14*	Body	0.90	(0.70, 1.17)	4.26E−01	0.92	(0.72, 1.19)	5.22E−01
cg13845521	21238886	*TTTY14*	Body	0.98	(0.74, 1.29)	8.76E−01	0.96	(0.72, 1.29)	7.62E−01
cg11816202	21239332	*TTTY14*	TSS200	1.16	(0.90, 1.50)	2.77E−01	1.17	(0.88, 1.53)	2.82E−01
cg15345074	21239461	*TTTY14*	TSS200	0.91	(0.69, 1.20)	5.00E−01	0.91	(0.69, 1.20)	5.25E−01
cg01988452	22736528	*EIF1AY*	TSS1500	0.99	(0.75, 1.30)	9.40E−01	1.03	(0.78, 1.36)	8.11E−01
cg13308744	22736584	*EIF1AY*	TSS1500	1.03	(0.80, 1.33)	8.13E−01	1.03	(0.78, 1.36)	8.44E−01
cg14467015	23757241	*TTTY13*	TSS1500	0.92	(0.70, 1.21)	5.49E−01	0.87	(0.65, 1.17)	3.46E−01
Weighted MRS				1.15	(0.72, 1.83)	5.57E−01	1.20	(0.75, 1.92)	4.48E−01

Note

Model 1 was adjusted for age, BMI, smoking status, and alcohol drinking status. Model 2 was additionally adjusted for education level, physical activity, and consumption of meat, vegetables, and fruits. Weighted MRS was calculated as the sum of coefficient multiplied by methylation level of each age‐related CpG.

## 3 DISCUSSION

To provide insight on age‐associated changes in DNA methylation on ChrY, we carried out a two‐stage investigation among 1,006 Chinese males aged 22.0–89.0 years old and identified 18 age‐related Y‐CpGs in peripheral blood. More importantly, among them, the methylation levels of cg03441493 and cg17816615 were significantly associated with all‐cause mortality risk of males. These results uncover the important role of ChrY methylation in male aging.

In the discovery stage, we observed that the methylation levels of 41 CpGs on ChrY were significantly associated with age at FDR < 0.05, of which 36 CpGs were hypermethylated with increasing age. Among them, 18 CpGs were validated to be associated with age in the validation stage, and 14 showed positive associations. Different from the reported hypomethylated age‐related methylation patterns with increasing age on autosomal chromosomes (Li, Christiansen et al., 2017; Marttila et al., 2015), our results suggested a tendency of hypermethylated Y‐CpGs with aging. This tendency was consistency with that reported by Lund et al and Vidaki et al (Lund et al., 2020; Vidaki et al., 2021). Lund et al analysed methylome data of 624 European men from four cohort studies and found seven age‐associated Y‐CpGs with FDR<0.05 in all four cohorts (Lund et al., 2020). One CpG, cg01707559 in *TBL1Y*, reported in our study, was also replicated by Lund et al's study. In addition, other 12 age‐related CpGs in *DDX3Y*, *TMSB4Y*, *NLGN4Y*, *TTTY14*, *EIF1AY*, and *TTTY13* found in the present study, were also reported to be associated with aging in at least one cohort in Lund et al's study. Furthermore, the methylation levels of three CpGs (cg17816615 in *DDX3Y*, cg13308744 in *EIF1AY*, and cg04691144 in *NLGN4Y*) in the current study were also associated with age in 1,057 males in the research carried out by Vidaki et al, suggesting the reliability and validity of the present results (Vidaki et al., 2021).

Cigarette smoking and alcohol drinking, as the important lifestyle factors, could cause epigenetic alterations in human genome, also in sex chromosomes (Alegria‐Torres et al., 2011). Klebaner et al found the significant relationships between smoking status and the methylation of two X‐CpGs (cg07764473 and cg21380860) in peripheral blood among 1,014 participants (Klebaner et al., 2016). It is plausible that smoking and alcohol drinking may influence the methylation level of Y‐CpGs. We found the methylation level of cg13845521 among these 18 CpGs showed suggestive significant association with the number of smoking pack‐year, and the methylation levels of cg13845521 and cg11816202, annotated to *TTTY14*, were associated with alcohol drinking status. However, smoking and alcohol drinking status could not modify the associations between these CpGs and age. Further studies with large sample size populations were warranted to validate these results.

One approach to functionally annotating CpGs is to analyse gene expression changes that may be relevant to the methylation of age‐associated CpGs. In the present study, we found that the methylation levels of cg13845521 and cg11816202 were negatively associated with the expression level of *TTTY14*. And the methylation levels at cg01988452 and cg13308744 were negatively associated with the expression level of *EIF1AY*. However, we did not find associations of other age‐related Y‐CpGs with the expression of their annotated genes. Linking the activity of age‐related CpGs with specific gene expression has proven to be difficult. Given the fact that the epigenetic state of cells in peripheral blood is heterogeneous, many age‐associated CpGs might not be related to gene expression (Horvath & Raj, 2018). In the future, transcriptomic and epigenetic sequencing at a single cell may help to establish the functional link between age‐related CpGs and annotated genes.

Although studying the age‐related changes in DNA methylation can help to assess the epigenetic regulation during human aging, it would be valuable to examine their associations with mortality risk, which is an objective measure of the overall population health (Lund et al., 2019). In males of the DFTJ sub‐cohort (mean age: 66.6 years old), the two age‐related Y‐CpGs, cg03441493 and cg17816615, were significantly associated with all‐cause mortality. The methylation level of cg03441493 that hypermethylated with age was positively associated with the hazard of death, while the methylation level of cg17816615 that hypomethylated with age, was negatively related to mortality. These findings seemed to be consistent with the general expectation that aging was related to increased risk of death. However, Lund et al found most of hypermethylated age‐related Y‐CpGs were associated with the decreased risk of mortality in LBC1921 study (*N* = 238, deaths = 151) (Lund et al., 2020). Given the fact that subjects in LBC1921 were octogenarians (aged 79.01–94.97 years old), this protective effect of ChrY methylation pattern may be attributed to subjects' successful aging. The heterogeneities in lifestyle habit and ethnic background may also explain the difference. The associations between age‐related Y‐CpGs and all‐cause mortality merit further investigation to provide clues for elucidating the underlying biology.

Current literatures in regard to the function of ChrY genes are limited. *TBL1Y*, a highly expressed gene in the prostate, is suggested to be critical in the cardiac differentiation and syndromic hearing loss (Di Stazio et al., 2019; Meyfour et al., 2017). Huang et al reported that the hypermethylation of *TBL1Y* was significantly associated with the risk of gastric cancer among 138 intestinal metaplasia patients in a longitudinal cohort study (Huang et al., 2018). *TMSB4Y*, which encodes an actin sequestering protein, acts as a candidate tumor suppressor and the overexpression of *TMSB4Y* results in the change of cell morphology with subsequent retardation of cell cycle progression (Wong et al., 2015). Utilizing in vitro study in prostate cancer cell PC‐3, Gong et al found that the expression of *NLGN4Y* negatively controlled cell proliferation and decreased cell migration through modifying GTPase activities (Gong et al., 2016). Three long noncoding genes (*TTTY13*, *TTTY14*, and *TTTY15*), located in the male‐specific region of the Y chromosome, are found to be involved in the prognosis of multiple cancers, such as gastric cancer and laryngeal squamous cell carcinoma (Cheng et al., 2019; Gong et al., 2020). All these findings support the hypothesis that age‐associated DNA methylation on ChrY in annotated genes may participate in tumorigenesis. In consideration that cancer is one of the age‐associated co‐morbidities, aging and tumorigenesis may share common biological process (Maegawa et al., 2017). However, the biological roles of age‐related CpGs on ChrY in aging process and mortality still need further investigations.

To our knowledge, this is the first study to detect age‐dependent Y‐CpGs in Chinese males. In the current study, we used methylome data across two‐stage populations to ensure reliability of results. We also found two age‐related Y‐CpGs, which were associated with all‐cause mortality, suggesting a critical role of ChrY in the aging process. However, several limitations should be noted. First, ChrY comprises about only 2% of the human genome. The number of Y‐CpGs on the Illumina Methylation 450K array (416 CpGs) and on the Illumina Methylation EPIC array (537 CpGs) are both less than 0.1% of the total number of CpGs on the arrays (485,512 CpGs and 866,554 CpGs, respectively), which means that CpGs for ChrY are highly underrepresented. In view of the limited numbers of Y‐CpGs, our results should be interpreted with caution. Sequencing technique will be required to detect more Y‐CpGs comprehensively in the future. Second, since this study included several male Chinese panels in the discovery stage, the heterogeneity of these populations may exist and the current results may not be generalized to other ethnic groups. However, meta‐analysis of the subset populations did not show heterogeneity and some previously reported age‐related Y‐CpGs in European males were also identified in the current study, supporting the reliability of the current findings. Third, the age span of study participants in the validation stage (42.5–87.5 years old) was shorter than that in the discovery stage (22.0–89.0 years old), which might reduce the repeatability of the results. However, Vidaki et al found that, in contrast to autosomal CpG‐based age predictors that were known to predict age with reduced accuracy in the elderly, the accuracy of DNA methylation on ChrY as the age predictor did not worsen with increased age (Vidaki et al., 2021), indicating that the impact of age span on the number of significant age‐related Y‐CpGs was limited. Further studies using large sample‐sized populations with comparable age span should be conducted to verify the results in the present study. Fourth, despite a lack of cross‐validation in the discovery stage, considering the limited sample sizes in three study panels, we combined all three panels together in the association analysis to improve the statistical power, which is a common approach in epidemiological studies with small sample sizes (Liu et al., 2021; Zhu et al., 2016). In addition, the gene expression profiles were only determined in the SY panel with limited sample size but not in subjects from the other panels, which might decrease the statistical power for discovering CpGs with regulation capacities. Lastly, the follow‐up time of DFTJ panel was relatively short and the number of death events was limited (*n* = 55, accounting for 9.4% in total). Further prospective studies with longer period are warranted to validate the association of age‐dependent Y‐CpGs with overall and cause‐specific mortality risk.

In conclusion, we identified 18 age‐related CpGs on ChrY and observed that two of them, cg03441493 and cg17816615, were associated with all‐cause mortality risk of Chinese males. Although our findings require further verification, these findings may gain insights into ChrY epigenetics and uncover the roles of ChrY epigenetic changes on male aging.

## 4 EXPERIMENTAL PROCEDURES

### 4.1 Study populations

We performed a two‐stage epigenetic analysis in a total of 1,006 Chinese males, in order to identify age‐related DNA methylation on ChrY. The discovery stage included a total of 419 healthy males from three panels, including 205 from the WHZH panel, 107 from the COW panel, and 107 from the SY panel. Detailed information regarding the participants in these panels has been described in the previous study (Li et al., 2021). Briefly, the WHZH panel included 180 Wuhan (WHZH‐Wuhan) residents and 103 Zhuhai (WHZH‐ Zhuhai) residents selected from the WHZH cohort at baseline (Song et al., 2014). Among these 283 participants, 205 males were included in the following analysis after quality control. The COW panel consisted of 144 healthy workers randomly selected from a coke oven plant in Wuhan, China (Li et al., 2012), and 107 male workers who passed the quality control were included in the following analysis. The SY panel contained a total of 144 healthy individuals who participated in the regular physical examinations at the Health Examination Center of Dongfeng Central Hospital in Shiyan, Hubei, China, during April and May of 2015 (Zhu et al., 2016). There were 107 males among these subjects and all passed quality control.

The validation stage was based on the DFTJ cohort study, which was an ongoing dynamic prospective cohort (Wang et al., 2013). A total of 38,295 subjects were recruited in 2013. Among them, those who had previous history of cancer, cardiovascular heart disease, or stroke (*n* = 10,254) and did not provide blood samples (*n* = 3,626) at baseline were excluded. A sub‐cohort of 1,399 participants among the left 24,415 subjects were randomly selected by age‐ and gender‐stratified sampling with an overall sampling rate of 5.8%. There were 602 males in the sub‐cohort subjects and 587 males passed the quality control.

In the DFTJ cohort, each subject had a unique medical insurance number and was tracked through the medical insurance system of the Dongfeng Motor Company for the cause‐specific mortality (Meng et al., 2020). Causes of death were confirmed by medical records from hospitals and death certificates from Centers for Diseases Control and Prevention. Mortality data were available until December 31, 2018 in the DFTJ cohort. We used the International Statistical Classification of Diseases and Related Health Problems Tenth Revision (ICD‐10) to classify the causes of death.

In the current study, each participant provided an informed consent and the study was approved by the Ethics Committee of Tongji Medical College, Huazhong University of Science and Technology.

### 4.2 Assessment of covariates

The general information of the study participants in both discovery and validation stages was collected through questionnaires and physical measurements at the baseline survey by trained staff. Subjects who had smoked >1 cigarette per day for >1 year were defined as current smokers; those who ever smoked and had quitted over half a year were defined as former smokers; otherwise, they were defined as never smokers. Smoking pack‐year was calculated as packs per day multiplied by years of smoking. Subjects who had drunk alcohol at least once a week for more than half a year were defined as current alcohol drinkers; those had ever drunk alcohol but quitted over half a year were defined as former alcohol drinkers; otherwise, they were defined as never alcohol drinkers. We combined current and former smokers into ever smokers, and combined current and former alcohol drinkers into ever drinkers (Wang et al., 2019). Weight and height were measured by trained examiners with participants standing without shoes. BMI was calculated as weight divided by height squared (kg/m^2^).

### 4.3 DNA methylation detection and quality control

Genome‐wide methylation assays for participants in the COW, WHZH, and SY panel were conducted by using Illumina Human Methylation 450K array and have been described previously (Li et al., 2021). Methylation of genomic DNA was quantified by using Illumina Human Methylation EPIC array in the DFTJ sub‐cohort. Briefly, DNA samples were extracted from peripheral whole blood by using the BioTeke Whole Blood DNA Extraction Kit (BioTeke, Beijing, China). Bisulfite conversion was performed by using the EZ DNA Methylation kit (Zymo Research, Orange, CA, USA) according to manufacturer's instructions. DNA methylation levels at >485,000 and >860,000 CpGs were quantified by using Infinium Human Methylation 450K BeadChip and Infinium Humen Methylation EPIC BeadChip (Illumina, Inc., Boston, USA), respectively.

Raw signal intensities were obtained by using the minfi package from IDAT files generated from iScan system (Illumina). Samples were removed if they: (1) were outliers in the test of Multidimensional Scaling; (2) had a missing rate>0.05 across probes; (3) were with individual‐call rate <0.98; (4) were gender mismatch. Then, samples were normalized internally only by their CpGs on ChrY (416 CpGs on Illumina 450K array and 537 CpGs on Illumina EPIC array). The CpG probes were removed if they: (1) had an overall sum of 10 or more cross‐reactive targets; (2) potentially contained or extended on SNPs with MAF>0.05 in the 1000 Genomes Project for Asian population (Chen et al., 2013); (3) had detection *p* > 0.01 among more than 5% samples. After filtering, methylation levels of 393 CpGs on Illumina 450K array and 520 CpGs on Illumina EPIC array were normalized by subset‐quantile within array normalization (Maksimovic et al., 2012). The *β* value, represented as the ratio of the signal intensity of the methylated probe to the total locus signal intensity, was calculated as M/M + U, with M and U for the methylated and unmethylated signal intensity, respectively. Probes were annotated using information provided by Illumina (genome build: GRCh37/hg19 genome assembly).

### 4.4 Gene expression detection in peripheral blood

The gene expression profiles for subjects in the SY panel were performed by using HumanHT‐12 v4 Expression BeadChip (Illumina, Inc.), and detailed description of the laboratory method had been published previously (Li, Zhu et al., 2017). Briefly, total RNA was extracted from peripheral blood within 2 h of collection using the TRIzol LS Reagent total RNA isolation kit (Life Technologies). Gene expression profiles were assayed using HumanHT‐12 v4 BeadChip (Illumina) according to standard protocols. Raw IDAT files were imported into the GenomeStudio v1.9.0, and quantile‐quantile normalization was used to normalize the signals (Dunning et al., 2007).

### 4.5 Statistical analyses

In order to describe the distribution of DNA methylation on ChrY, we compared the inter‐quartile range of *β* values of CpGs on autosomal chromosome (Chr22), ChrX, and ChrY probes by using Kruskal–Wallis rank sum test, which assumed that normality was not met. Histograms of *β* values of ChrY CpGs were drawn among different panels in the current study. To eliminate outliers and archive a normal distribution, *β* value of each CpG on ChrY was inverse normal transformed (INT, to a normal distribution with a mean value of 0 and a standard deviation of 1).

The associations between age and methylation levels of all CpGs on ChrY were analysed by using linear regression model. For participants in the discovery stage, the regression model was adjusted for BMI, smoking status, alcohol drinking status, major leukocyte compositions (neutrophils, lymphocytes as well as intermediate cells, which were defined as the sum of monocytes, eosinophils, and basophils), and experimental batch; the CpGs with FDR <0.05 were considered as statistically significant. Chromosomal ideogram was visualized using PhenoGram software (http://visualization.ritchielab.org/phenograms/plot). In the validation stage, the regression model was adjusted for BMI, smoking status, alcohol drinking status, and major leukocyte compositions; the CpGs with *p* < 0.05 were considered as statistically significant. Given that two types of Illumina methylation arrays were used in the present study, results from the discovery and validation stages were combined by using inverse‐variance weighted fixed‐effect meta‐analysis (FDR < 0.05 was used as a threshold to define meta‐analysis significance).

To evaluate the modification effects of smoking and alcohol drinking status, we firstly tested the relationships of DNA methylation with smoking pack‐year and alcohol drinking status. Then, stratification analyses were conducted according to smoking and alcohol drinking status. Additionally, we tested the joint effect of significant CpGs based on weighted MRS. For all ChrY CpGs with significant association with age, the weighted MRS was calculated as the sum of their coefficients with age multiplied by methylation levels of corresponding CpGs (Huls & Czamara, 2020). The calculation formula was listed as follows: weighted MRS = *β_1_* X_1_ + *β_2_* X_2_+_…_+*β_n_* X_n_, where *β* represented the estimated regression coefficient of the CpG site derived from the linear regression analysis, and X represented the methylation level of the CpG site.

In addition, we annotated CpGs to the nearest genes by using the annotation files provided by Illumina. The correlations between methylation levels of ChrY CpGs and expression levels of their corresponding genes (both values were INT) were assessed by using linear regression models, with adjustment for age, smoking status, alcohol drinking status, and BMI.

Furthermore, since the males in the DFTJ sub‐cohort (those in the validation stage) had available follow‐up information, we performed survival analysis to estimate the associations between the methylation levels of age‐related ChrY CpGs and total mortality risk among this population by using Cox proportional hazard model. Model 1 was adjusted for age, BMI, smoking status, and alcohol drinking status; model 2 was additionally adjusted for education level, physical activity, and consumption of meat, vegetables, and fruits. Variables adjusted in the models were selected according to the previous study (Meng et al., 2020). All analyses were performed by using R version 3.6.1, except for the meta‐analysis, which was performed by using METAL at http://csg.sph.umich.edu/abecasis/metal/.

## CONFLICT OF INTEREST

The authors declared no competing interests.

## AUTHOR CONTRIBUTIONS

G.L. and H.G. conceived this study, analysed the data, interpreted the findings, and drafted this manuscript. All authors helped the data collection, commented on the manuscript, and approved the submitted version.

## ETHICS APPROVAL AND CONSENT TO PARTICIPATE

All participants were provided informed consents, and this work has received approval for research ethics from the Ethics Committee of Tongji Medical College, Huazhong University of Science and Technology. A proof/certificate of approval is available upon request (no. S335).

## Supporting information

Supplementary Material

## Data Availability

The datasets used and/or analyzed during the current study are available from the corresponding author on reasonable request.
