# Supplementary material for: Age‐related DNA methylation on Y chromosome and their associations with total mortality among Chinese males
Source: Aging Cell. 2022 Feb 4;21(3):e13563. doi: 10.1111/acel.13563 (PMC8920452; doi:10.1111/acel.13563)
Supplement: Supplementary file 1 — Supplementary Material [file ACEL-21-e13563-s001.docx]

**Table of Contents**

**Table S1.** Associations between methylation levels of 41 CpGs on ChrY and age in the discovery and validation stages.

**Table S2.** Meta-analysis for the associations of DNA methylation levels of 18 CpGs on ChrY with smoking pack-year and alcohol drinking status.

**Table S3.** Meta-analysis for the associations between the methylation levels of 18 CpGs on ChrY and age when stratified by smoking status.

**Table S4.** Meta-analysis for the associations between the methylation levels of 18 CpGs on ChrY and age when stratified by alcohol drinking status.

**Table S5.** Methylation-gene expression correlations for age-related CpGs on ChrY in SY panel.

**Figure S1.** Cumulative density distribution of inter-quantile range (IQR) for autosomal (black), ChrX (blue) and ChrY (red) probes in the discovery stage (A) and validation stage (B).

**Figure S2.** Distribution of β values of ChrY CpGs for study participants in each study panel.

**Figure S3.** Physical locations of age-related CpGs on ChrY.

**Figure S4.** Association of weighted methylation risk score on ChrY with age in each study panel. Trend line was fitted by linear regression model.

**Figure S5.** Correlations between age and the expression levels of *TTTY14* and *EIF1AY*.

A: *TTTY14* probe ILMN_2143383; B: *EIF1AY* probe ILMN_1755537; C: *EIF1AY* probe ILMN_2228976.

**Table S1. Associations between methylation levels of 41 CpGs on ChrY and age in the discovery and validation stages.**

| **CpG** | **Position** | **Gene** | **Relation to Gene** | **Discovery stage** | | | |  | **Validation stage** | | |
| --- | --- | --- | --- | --- | --- | --- | --- | --- | --- | --- | --- |
|  |  |  |  | **β** | **SE** | ***P*** | **FDR** |  | **β** | **SE** | ***P*** |
| cg04169747 | 2655740 | *SRY* | 5’UTR | 1.97 | 0.64 | 2.37E-03 | **2.59E-02** |  | *-* | *-* | - |
| cg27636129 | 2655927 | *SRY* | TSS200 | 2.14 | 0.60 | 4.41E-04 | **6.19E-03** |  | *-* | *-* | - |
| cg09595415 | 2655942 | *SRY* | TSS200 | 2.43 | 0.60 | 6.18E-05 | **1.43E-03** |  | 0.50 | 0.27 | 6.71E-02 |
| cg15563434 | 2657879 |  |  | 2.49 | 0.67 | 2.56E-04 | **4.13E-03** |  | 0.51 | 0.27 | 5.78E-02 |
| cg07939587 | 2657936 |  |  | 2.38 | 0.64 | 2.44E-04 | **4.13E-03** |  | 0.32 | 0.27 | 2.27E-01 |
| cg03767353 | 2658057 |  |  | 2.07 | 0.57 | 3.25E-04 | **4.91E-03** |  | 0.36 | 0.27 | 1.74E-01 |
| cg10465579 | 4867963 | *PCDH11Y* | TSS1500 | 2.17 | 0.63 | 5.97E-04 | **8.09E-03** |  | 0.38 | 0.31 | 2.14E-01 |
| cg15295597 | 4868368 | *PCDH11Y* | 5’UTR | 2.88 | 0.67 | 2.39E-05 | **6.25E-04** |  | 0.51 | 0.28 | 7.34E-02 |
| cg02494853 | 4868397 | *PCDH11Y* | 5’UTR | 1.78 | 0.63 | 4.89E-03 | **4.69E-02** |  | 0.26 | 0.28 | 3.64E-01 |
| cg02432075 | 4870822 | *PCDH11Y* | 5’UTR | 3.23 | 0.64 | 6.21E-07 | **2.71E-05** |  | 0.28 | 0.30 | 3.53E-01 |
| cg02002345 | 6777855 | *TBL1Y* | TSS1500 | 1.71 | 0.60 | 4.67E-03 | **4.59E-02** |  | 0.08 | 0.28 | 7.77E-01 |
| cg02839557 | 6778641 | *TBL1Y* | TSS200 | 2.00 | 0.61 | 1.13E-03 | **1.30E-02** |  | 0.33 | 0.28 | 2.28E-01 |
| cg01707559 | 6778695 | *TBL1Y* | TSS200 | 3.51 | 0.66 | 1.95E-07 | **1.27E-05** |  | 0.67 | 0.27 | **1.38E-02** |
| cg15197499 | 6778939 | *TBL1Y* | 1stExon | 2.04 | 0.63 | 1.40E-03 | **1.58E-02** |  | 0.26 | 0.27 | 3.30E-01 |
| cg02407581 | 8553302 | *TTTY18* | TSS1500 | 2.23 | 0.60 | 2.41E-04 | **4.13E-03** |  | *-* | *-* | - |
| cg14210405 | 9930240 |  |  | 1.79 | 0.59 | 2.49E-03 | **2.64E-02** |  | 0.21 | 0.27 | 4.47E-01 |
| cg03441493 | 14074417 |  |  | 2.08 | 0.56 | 2.63E-04 | **4.13E-03** |  | 0.55 | 0.27 | **3.82E-02** |
| cg09093035 | 14074689 |  |  | 3.09 | 0.55 | 4.18E-08 | **3.29E-06** |  | 0.94 | 0.26 | **3.80E-04** |
| cg13851368 | 14074873 |  |  | 2.60 | 0.62 | 3.30E-05 | **8.12E-04** |  | 0.42 | 0.27 | 1.21E-01 |
| cg03258315 | 14077975 |  |  | 2.31 | 0.69 | 9.58E-04 | **1.21E-02** |  | 0.62 | 0.27 | **2.03E-02** |
| cg08596608 | 14533505 |  |  | 2.19 | 0.57 | 1.59E-04 | **3.29E-03** |  | 0.31 | 0.29 | 2.87E-01 |
| cg09730640 | 14533708 |  |  | 3.36 | 0.56 | 3.33E-09 | **3.27E-07** |  | 0.86 | 0.28 | **2.63E-03** |
| cg25032547 | 14773536 | *TTTY15* | TSS1500 | 2.14 | 0.58 | 2.23E-04 | **4.13E-03** |  | 0.55 | 0.27 | **4.61E-02** |
| cg17816615 | 15019332 | *DDX3Y* | Body | -2.09 | 0.59 | 4.09E-04 | **5.96E-03** |  | -0.95 | 0.28 | **6.05E-04** |
| cg26198148 | 15814685 | *TMSB4Y* | TSS1500 | 2.28 | 0.61 | 2.02E-04 | **3.96E-03** |  | 0.95 | 0.28 | **6.20E-04** |
| cg06247034 | 16634255 | *NLGN4Y* | TSS1500 | 2.16 | 0.56 | 1.47E-04 | **3.22E-03** |  | 0.52 | 0.27 | 5.96E-02 |
| cg04691144 | 16634373 | *NLGN4Y* | TSS1500 | 4.62 | 0.65 | 6.74E-12 | **8.83E-10** |  | 0.70 | 0.30 | **1.75E-02** |
| cg02233183 | 16634382 | *NLGN4Y* | TSS1500 | 4.72 | 0.67 | 6.08E-12 | **8.83E-10** |  | *-* | *-* | - |
| cg27265812 | 16634594 | *NLGN4Y* | Body | 2.09 | 0.61 | 6.17E-04 | **8.09E-03** |  | 0.25 | 0.28 | 3.77E-01 |
| cg27443332 | 16634795 | *NLGN4Y* | Body | 2.52 | 0.57 | 1.03E-05 | **3.00E-04** |  | 1.11 | 0.28 | **1.05E-04** |
| cg27214488 | 16635501 | *NLGN4Y* | TSS200 | 3.20 | 0.66 | 2.03E-06 | **7.26E-05** |  | *-* | *-* | - |
| cg03706273 | 16635745 | *NLGN4Y* | 1stExon | 3.41 | 0.67 | 5.82E-07 | **2.71E-05** |  | 0.61 | 0.29 | **3.36E-02** |
| cg03244189 | 21238472 | *TTTY14* | Body | 2.06 | 0.63 | 1.11E-03 | **1.30E-02** |  | 0.67 | 0.28 | **1.79E-02** |
| cg13845521 | 21238886 | *TTTY14* | Body | 2.89 | 0.55 | 3.03E-07 | **1.70E-05** |  | 0.78 | 0.28 | **6.09E-03** |
| cg11816202 | 21239332 | *TTTY14* | TSS200 | 2.56 | 0.57 | 8.43E-06 | **2.76E-04** |  | 0.68 | 0.28 | **1.70E-02** |
| cg15345074 | 21239461 | *TTTY14* | TSS200 | 1.67 | 0.58 | 4.37E-03 | **4.41E-02** |  | 0.59 | 0.27 | **3.12E-02** |
| cg25756647 | 21728575 | *CYorf15A* | TSS1500 | 2.49 | 0.56 | 1.07E-05 | **3.00E-04** |  | 0.32 | 0.27 | 2.32E-01 |
| cg01988452 | 22736528 | *EIF1AY* | TSS1500 | -2.68 | 0.55 | 1.71E-06 | **6.72E-05** |  | -1.36 | 0.26 | **3.72E-07** |
| cg13308744 | 22736584 | *EIF1AY* | TSS1500 | -4.20 | 0.53 | 1.48E-14 | **5.83E-12** |  | -0.97 | 0.27 | **3.25E-04** |
| cg10172760 | 22736833 | *EIF1AY* | TSS1500 | -1.66 | 0.57 | 3.86E-03 | **3.99E-02** |  | *-* | *-* | - |
| cg14467015 | 23757241 | *TTTY13* | TSS1500 | -2.07 | 0.63 | 1.11E-03 | **1.30E-02** |  | -0.72 | 0.26 | **6.88E-03** |

**Note:** Association analyses were performed by using linear regression models, with inverse-normal transformed DNA methylation β values as the independent variables, age as the dependent variable, with adjustment for BMI, smoking status, drinking status, major leukocyte compositions, and experimental batches.

| **Table S2. Meta-analysis for the associations of DNA methylation levels of 18 CpGs on ChrY with smoking pack-year and alcohol drinking status.** | | | | | | | | | | |
| --- | --- | --- | --- | --- | --- | --- | --- | --- | --- | --- |
| **CpG** | **Position** | **Mapped Gene** | **Relation to Gene** | **Smoking pack-year ^a^** | | |  | **Alcohol drinking status ^b^** | | |
|  |  |  |  | **β** | **se** | ***P*** |  | **β** | **se** | ***P*** |
| cg01707559 | 6778695 | *TBL1Y* | TSS200 | -0.01 | 0.02 | 7.66E-01 |  | -0.02 | 0.06 | 6.80E-01 |
| cg03441493 | 14074417 |  |  | -0.01 | 0.02 | 5.24E-01 |  | 0.10 | 0.06 | 1.03E-01 |
| cg09093035 | 14074689 |  |  | 0.01 | 0.02 | 7.90E-01 |  | -0.02 | 0.06 | 7.66E-01 |
| cg03258315 | 14077975 |  |  | 0.02 | 0.02 | 2.55E-01 |  | 0.02 | 0.06 | 6.82E-01 |
| cg09730640 | 14533708 |  |  | 0.03 | 0.02 | 7.82E-02 |  | -0.08 | 0.06 | 1.73E-01 |
| cg25032547 | 14773536 | *TTTY15* | TSS1500 | 0.01 | 0.02 | 7.85E-01 |  | -0.04 | 0.06 | 5.28E-01 |
| cg17816615 | 15019332 | *DDX3Y* | Body | -0.02 | 0.02 | 2.29E-01 |  | -0.07 | 0.06 | 2.84E-01 |
| cg26198148 | 15814685 | *TMSB4Y* | TSS1500 | 0.01 | 0.02 | 5.58E-01 |  | -0.04 | 0.06 | 5.29E-01 |
| cg04691144 | 16634373 | *NLGN4Y* | TSS1500 | -0.004 | 0.01 | 7.85E-01 |  | 0.02 | 0.05 | 7.24E-01 |
| cg27443332 | 16634795 | *NLGN4Y* | Body | -0.01 | 0.02 | 4.94E-01 |  | -0.01 | 0.06 | 8.58E-01 |
| cg03706273 | 16635745 | *NLGN4Y* | 1stExon | 0.01 | 0.02 | 7.35E-01 |  | -0.10 | 0.06 | 6.72E-02 |
| cg03244189 | 21238472 | *TTTY14* | Body | 0.02 | 0.02 | 1.95E-01 |  | -0.11 | 0.06 | 5.26E-02 |
| cg13845521 | 21238886 | *TTTY14* | Body | 0.04 | 0.02 | **2.67E-02** |  | -0.19 | 0.06 | **2.20E-03** |
| cg11816202 | 21239332 | *TTTY14* | TSS200 | 0.01 | 0.02 | 4.24E-01 |  | -0.15 | 0.06 | **1.24E-02** |
| cg15345074 | 21239461 | *TTTY14* | TSS200 | 0.01 | 0.02 | 5.89E-01 |  | -0.07 | 0.06 | 2.38E-01 |
| cg01988452 | 22736528 | *EIF1AY* | TSS1500 | -0.01 | 0.02 | 5.90E-01 |  | 0.003 | 0.06 | 9.67E-01 |
| cg13308744 | 22736584 | *EIF1AY* | TSS1500 | -0.01 | 0.02 | 5.99E-01 |  | -0.09 | 0.06 | 1.29E-01 |
| cg14467015 | 23757241 | *TTTY13* | TSS1500 | 0.01 | 0.02 | 5.42E-01 |  | 0.01 | 0.06 | 9.14E-01 |
| Note: ^a^ Association analysis was performed by linear regression model, with smoking pack-year as the independent variable, inverse normal transformed DNA methylation β value as the dependent variable, with adjustment for age, BMI, alcohol drinking status, major leukocyte compositions and experiment batch (only in the discovery stage).  ^b^ Association analysis was performed by linear regression model, with alcohol drinking status as the independent variable, inverse-normal transformed DNA methylation β value as the dependent variable, with adjustment for age, BMI, smoking status, major leukocyte compositions and experiment batch (only in the discovery stage).  The results from two stages were combined by using a fixed-effect meta-analysis. | | | | | | | | | | |

| **Table S3. Meta-analysis for the associations between the methylation levels of 18 CpGs on ChrY and age when stratified by smoking status.** | | | | | | | | |
| --- | --- | --- | --- | --- | --- | --- | --- | --- |
| **CpG** | **Ever smokers (n=620)** | | |  | **Never smokers (n=386)** | | | ***P*_interaction_** |
|  | **β** | **SE** | ***P*** |  | **β** | **SE** | ***P*** |  |
| cg01707559 | 0.62 | 0.30 | **3.92E-02** |  | 1.96 | 0.45 | **1.22E-05** | 9.80E-03 |
| cg03441493 | 0.97 | 0.29 | **8.36E-04** |  | 0.60 | 0.43 | 1.62E-01 | 6.22E-01 |
| cg09093035 | 1.60 | 0.29 | **2.71E-08** |  | 0.88 | 0.42 | **3.70E-02** | 2.28E-01 |
| cg03258315 | 0.70 | 0.30 | **1.91E-02** |  | 1.26 | 0.44 | **4.05E-03** | 7.52E-01 |
| cg09730640 | 1.52 | 0.30 | **4.82E-07** |  | 1.42 | 0.45 | **1.72E-03** | 3.54E-01 |
| cg25032547 | 0.74 | 0.29 | **1.06E-02** |  | 1.43 | 0.46 | **1.89E-03** | 6.77E-01 |
| cg17816615 | -0.96 | 0.30 | **1.51E-03** |  | -1.23 | 0.44 | **5.28E-03** | 6.70E-01 |
| cg26198148 | 1.03 | 0.30 | **6.34E-04** |  | 1.60 | 0.46 | **4.60E-04** | 9.77E-01 |
| cg04691144 | 1.34 | 0.32 | **3.00E-05** |  | 1.69 | 0.48 | **4.67E-04** | 7.52E-01 |
| cg27443332 | 1.28 | 0.31 | **3.30E-05** |  | 1.77 | 0.45 | **8.05E-05** | 8.29E-01 |
| cg03706273 | 0.61 | 0.32 | **5.93E-02** |  | 1.61 | 0.46 | **4.62E-04** | 8.82E-02 |
| cg03244189 | 0.68 | 0.31 | **2.99E-02** |  | 1.24 | 0.46 | **6.85E-03** | 4.57E-01 |
| cg13845521 | 0.86 | 0.30 | **4.71E-03** |  | 1.97 | 0.45 | **1.25E-05** | 3.25E-01 |
| cg11816202 | 0.86 | 0.31 | **5.16E-03** |  | 1.37 | 0.45 | **2.24E-03** | 6.64E-01 |
| cg15345074 | 0.93 | 0.30 | **1.90E-03** |  | 0.59 | 0.44 | 1.83E-01 | 3.87E-01 |
| cg01988452 | -1.18 | 0.29 | **4.75E-05** |  | -2.29 | 0.42 | **5.47E-08** | 1.76E-02 |
| cg13308744 | -1.33 | 0.29 | **3.98E-06** |  | -2.18 | 0.42 | **2.26E-07** | 4.97E-02 |
| cg14467015 | -1.05 | 0.29 | **3.22E-04** |  | -0.91 | 0.44 | **3.93E-02** | 3.17E-01 |
| Note: Association analysis was performed by using linear regression model in each stratum, with inverse-normal transformed DNA methylation β value as the independent variable, age as the dependent variable, with adjustment for alcohol drinking status, BMI, and major leukocyte compositions and experiment batch (only in the discovery stage).  The results from two stages were combined by using a fixed-effect meta-analysis. | | | | | | | | |

| **Table S4. Meta-analysis for the associations between the methylation levels of 18 CpGs on ChrY and age when stratified by alcohol drinking status.** | | | | | | | | |
| --- | --- | --- | --- | --- | --- | --- | --- | --- |
| **CpG** | **Ever alcohol drinkers (n=506)** | | |  | **Never alcohol drinkers (n=500)** | | | ***P*_interaction_** |
|  | **β** | **SE** | ***P*** |  | **β** | **SE** | ***P*** |  |
| cg01707559 | 1.02 | 0.32 | **1.38E-03** |  | 0.93 | 0.40 | **2.17E-02** | 8.95E-01 |
| cg03441493 | 1.04 | 0.31 | **7.64E-04** |  | 0.62 | 0.38 | 1.03E-01 | 3.40E-01 |
| cg09093035 | 1.26 | 0.31 | **3.88E-05** |  | 1.43 | 0.38 | **1.72E-04** | 9.80E-01 |
| cg03258315 | 0.26 | 0.32 | 4.20E-01 |  | 1.66 | 0.39 | **2.12E-05** | 5.42E-02 |
| cg09730640 | 1.68 | 0.33 | **2.24E-07** |  | 1.10 | 0.40 | **6.31E-03** | 3.57E-01 |
| cg25032547 | 0.66 | 0.32 | **4.06E-02** |  | 1.00 | 0.38 | **9.13E-03** | 7.60E-01 |
| cg17816615 | -1.08 | 0.32 | **8.27E-04** |  | -1.04 | 0.40 | **9.09E-03** | 7.41E-01 |
| cg26198148 | 1.26 | 0.33 | **1.24E-04** |  | 1.11 | 0.40 | **5.55E-03** | 5.52E-01 |
| cg04691144 | 1.13 | 0.34 | **1.01E-03** |  | 1.62 | 0.43 | **1.80E-04** | 1.28E-01 |
| cg27443332 | 1.36 | 0.33 | **2.87E-05** |  | 1.39 | 0.41 | **7.28E-04** | 5.31E-01 |
| cg03706273 | 1.35 | 0.34 | **8.20E-05** |  | 0.78 | 0.42 | 6.57E-02 | 8.86E-01 |
| cg03244189 | 1.05 | 0.33 | **1.44E-03** |  | 0.62 | 0.41 | 1.30E-01 | 9.77E-01 |
| cg13845521 | 1.50 | 0.32 | **2.73E-06** |  | 0.89 | 0.41 | **2.82E-02** | 3.66E-01 |
| cg11816202 | 0.84 | 0.33 | **1.06E-02** |  | 1.28 | 0.39 | **1.10E-03** | 2.88E-01 |
| cg15345074 | 0.71 | 0.32 | **2.83E-02** |  | 0.92 | 0.39 | **1.65E-02** | 5.16E-01 |
| cg01988452 | -1.41 | 0.31 | **4.34E-06** |  | -2.04 | 0.37 | **5.01E-08** | 3.11E-01 |
| cg13308744 | -1.52 | 0.31 | **1.07E-06** |  | -1.83 | 0.38 | **1.27E-06** | 9.38E-01 |
| cg14467015 | -0.91 | 0.32 | **3.94E-03** |  | -0.93 | 0.39 | **1.60E-02** | 4.47E-01 |
| Note: Association analyses were performed by using linear regression models in each stratum, with inverse-normal transformed DNA methylation β value as the independent variable, age as the dependent variable, with adjustment for smoking status, BMI, and major leukocyte compositions and experiment batch (only in the discovery stage).  The results from two stages were combined by using a fixed-effect meta-analysis. | | | | | | | | |

| **Table S5. Methylation-gene expression correlations for age-related CpGs on ChrY in SY panel.** | | | | | | |
| --- | --- | --- | --- | --- | --- | --- |
| **CpG** | **Transcript information** | |  | **CpG-expression association** | | |
|  | **Probe** | **Gene** |  | **β (SE)** | ***P*** | **Expression rate** |
| cg01707559 | ILMN_2317524 | *TBL1Y* |  | -0.0001 (0.10) | 9.99E-01 | 68.06% |
| cg25032547 | ILMN_2077896 | *TTTY15* |  | 0.08 (0.10) | 3.93E-01 | 88.89% |
| cg17816615 | ILMN_1732039 | *DDX3Y* |  | 0.06 (0.10) | 5.25E-01 | 85.42% |
| cg26198148 | ILMN_1776195 | *TMSB4Y* |  | -0.09 (0.10) | 3.81E-01 | 81.94% |
| cg04691144 | ILMN_2210199 | *NLGN4Y* |  | 0.01 (0.08) | 8.63E-01 | 40.28% |
| cg27443332 | ILMN_2210199 | *NLGN4Y* |  | -0.02 (0.08) | 8.10E-01 | 40.28% |
| cg03706273 | ILMN_2210199 | *NLGN4Y* |  | 0.01 (0.08) | 9.29E-01 | 40.28% |
| cg03244189 | ILMN_2143383 | *TTTY14* |  | -0.14 (0.10) | 1.37E-01 | 84.03% |
| cg13845521 | ILMN_2143383 | *TTTY14* |  | -0.20 (0.10) | **4.35E-02** | 84.03% |
| cg11816202 | ILMN_2143383 | *TTTY14* |  | -0.21 (0.09) | **3.06E-02** | 84.03% |
| cg15345074 | ILMN_2143383 | *TTTY14* |  | -0.13 (0.10) | 2.12E-01 | 84.03% |
| cg01988452 | ILMN_1755537 | *EIF1AY* |  | -0.25 (0.09) | **1.02E-02** | 97.22% |
|  | ILMN_2228976 | *EIF1AY* |  | -0.28 (0.09) | **3.83E-03** | 93.06% |
| cg13308744 | ILMN_1755537 | *EIF1AY* |  | -0.31 (0.10) | **3.02E-03** | 97.22% |
|  | ILMN_2228976 | *EIF1AY* |  | -0.34 (0.10) | **1.14E-03** | 93.06% |
| cg14467015 | ILMN_2080306 | *TTTY13* |  | 0.12 (0.08) | 1.49E-01 | 32.64% |
|  | ILMN_2080309 | *TTTY13* |  | 0.11 (0.08) | 1.69E-01 | 40.28% |
| **Note:** Methylation-expression correlations were calculated using linear regressions in which inverse normal transformed expression values were regressed on inverse normal transformed methylation values, with adjustment for age, smoking status, alcohol drinking status and BMI. The probes with signal detection proportion <0.3 were excluded in the current analysis. | | | | | | |


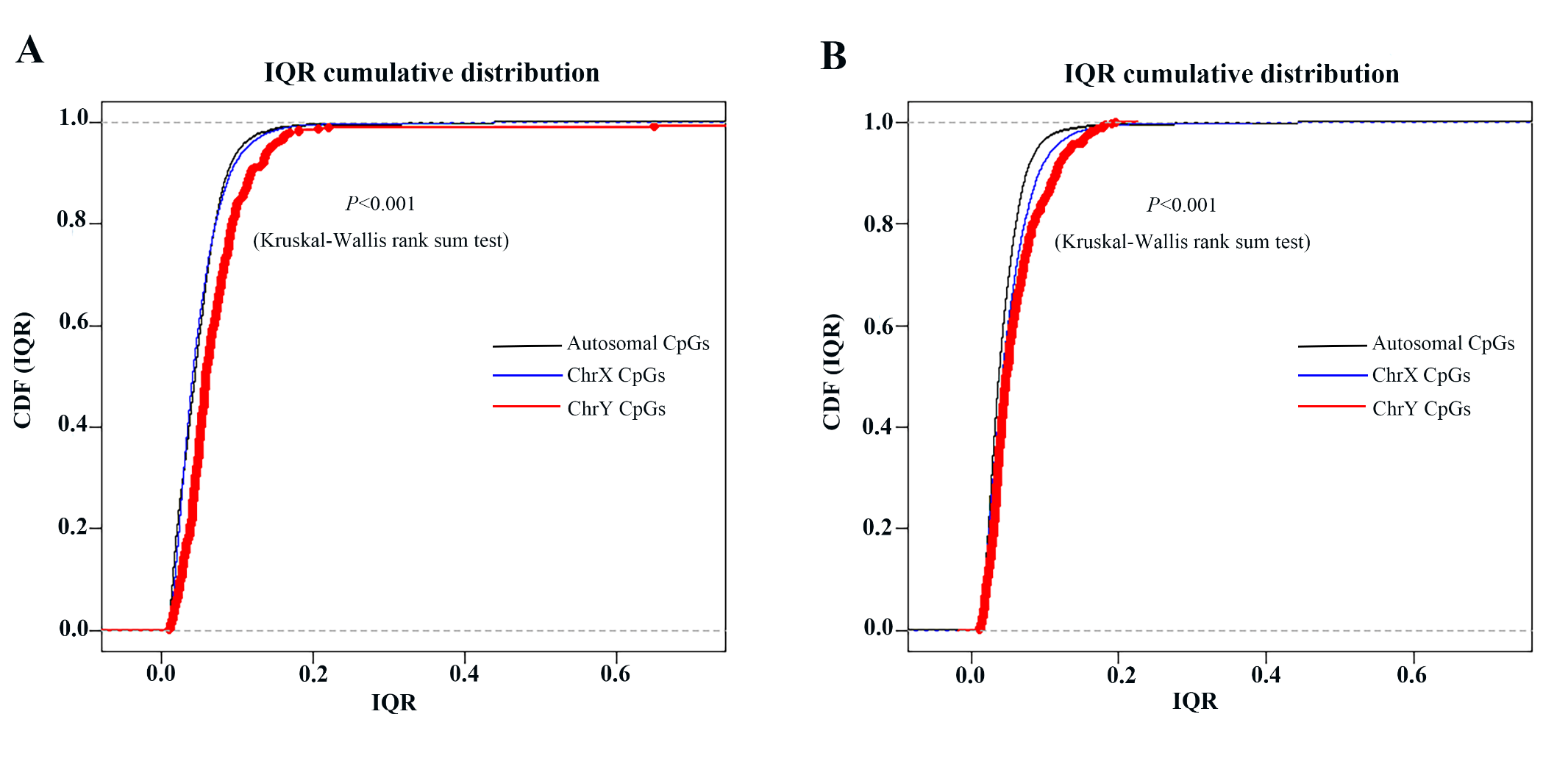


**Figure S1.** **Cumulative density distribution of inter-quantile range (IQR) for autosomal (black), ChrX (blue) and ChrY (red) probes in the discovery stage (A) and validation stage (B).**


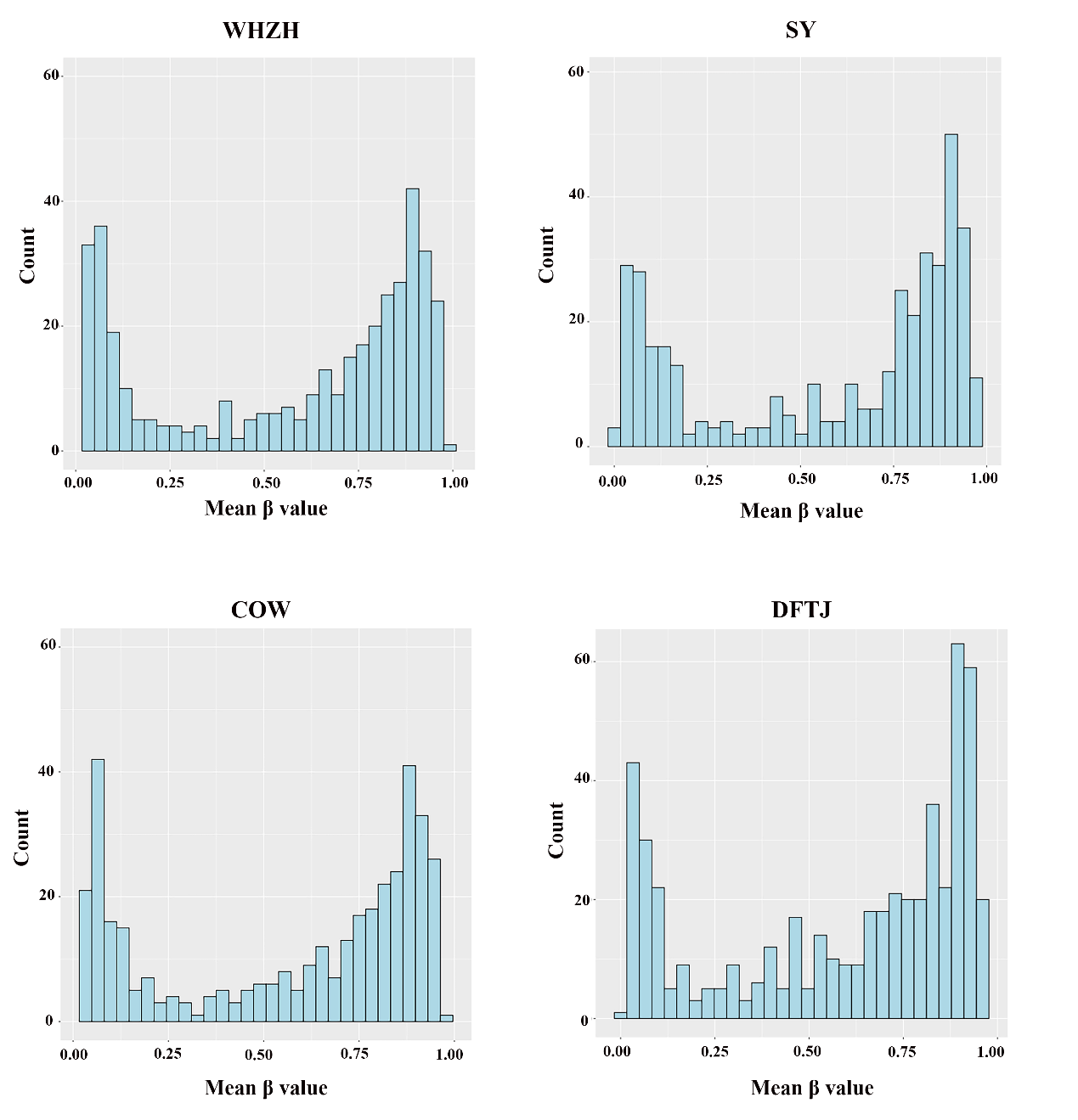


**Figure S2. Distribution of β values of ChrY CpGs for study participants in each study panel.**


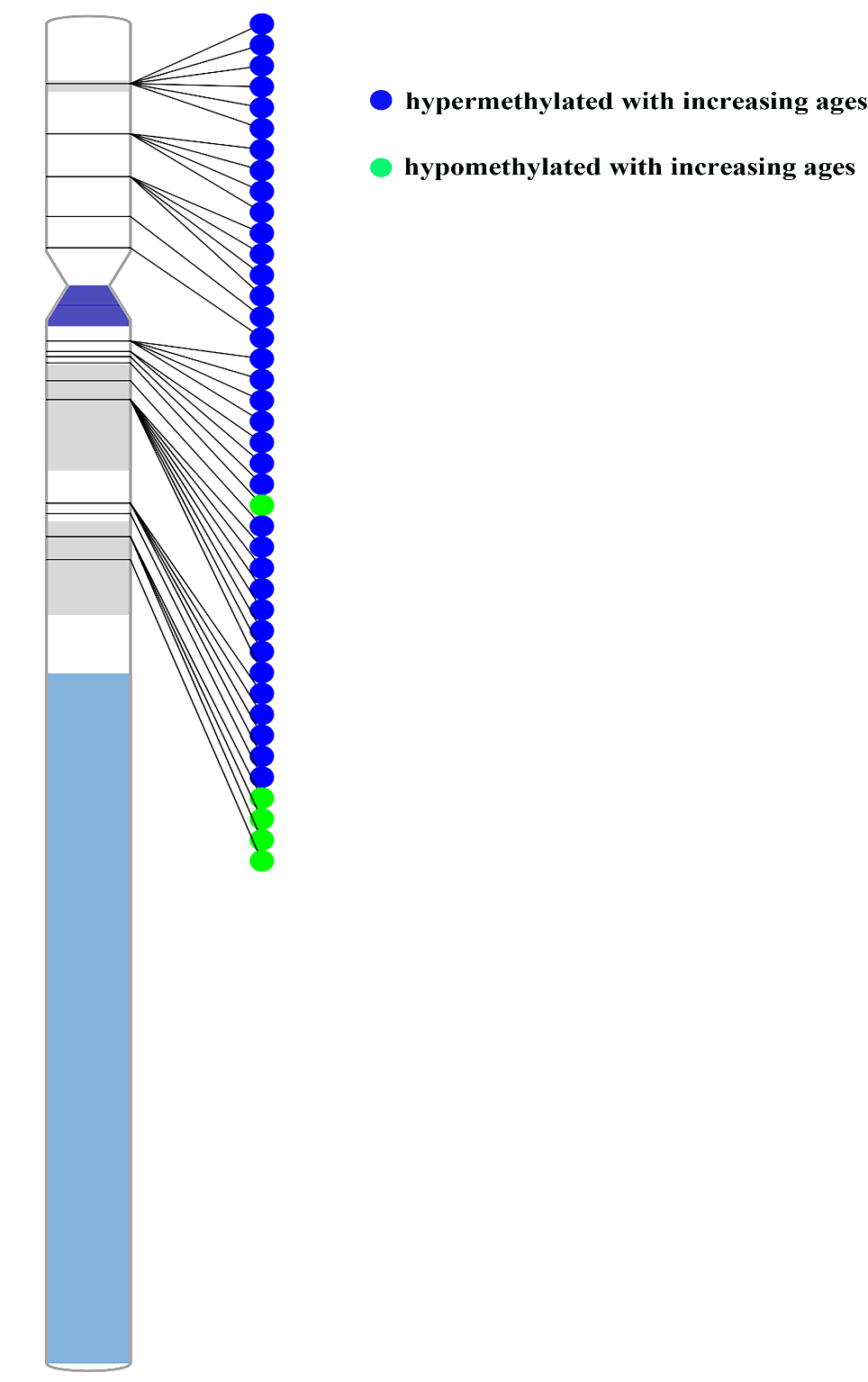


**Figure S3. Physical locations of age-related CpGs on ChrY.**

Notes: Genomic locations (black lines) are shown separately for age-related CpGs identified in the discovery stage where methylation increases (hyper), and decreases (hypo) with advancing age. Cytobands are indicated in blue and grey coloring.


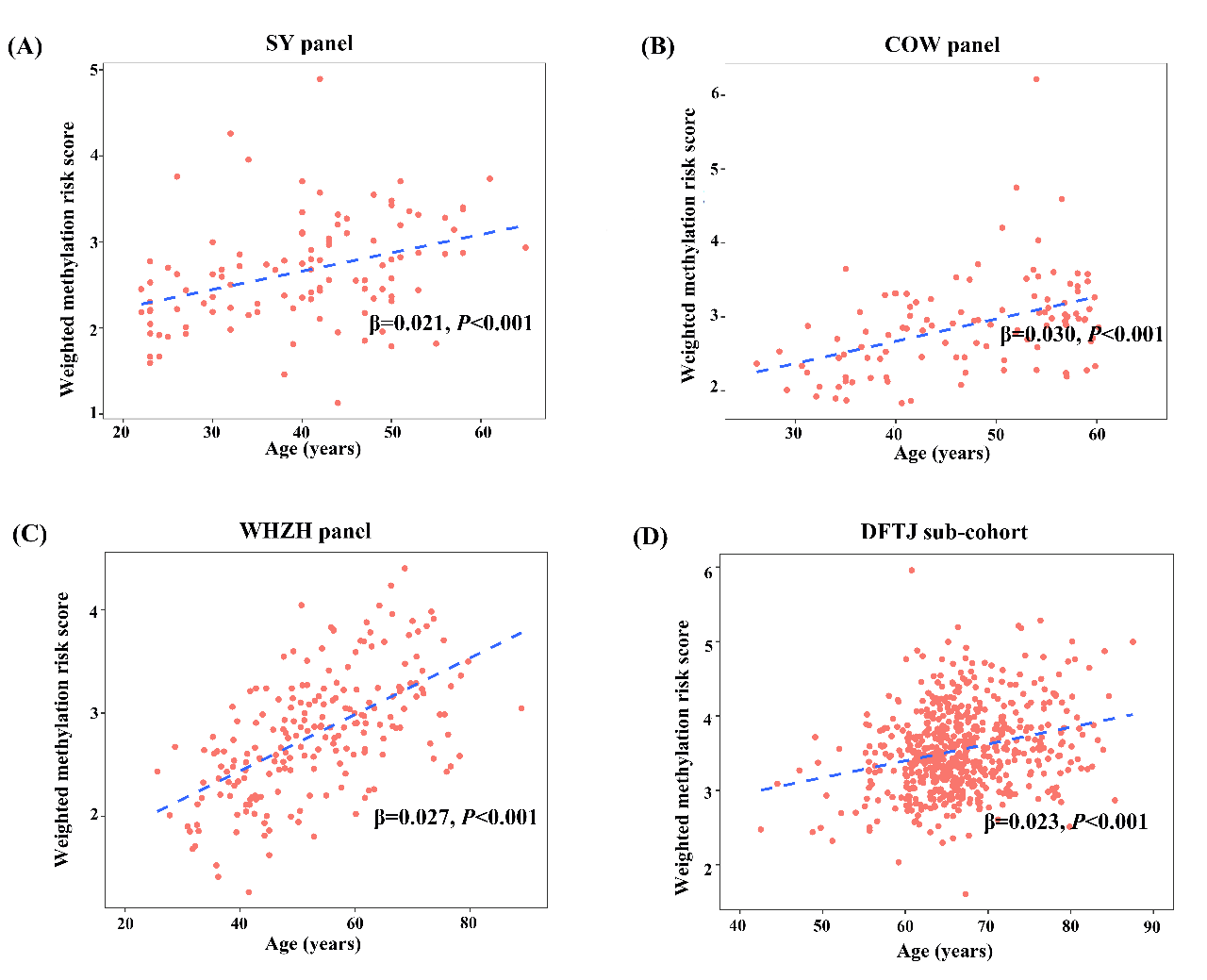


**Figure S4. Association of weighted methylation risk score on ChrY with age in each study panel. Trend line was fitted by linear regression model.**

**
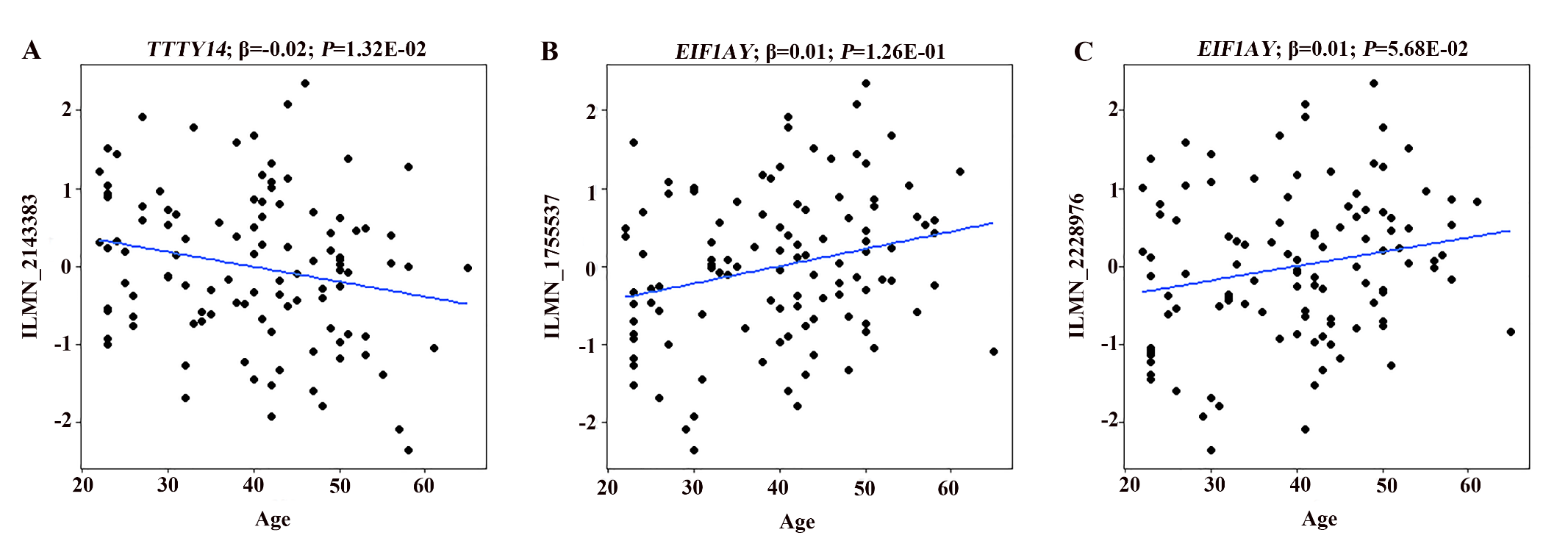
**

**Figure S5. Correlations between age and the expression levels of *TTTY14* and *EIF1AY*.**

A: *TTTY14* probe ILMN_2143383; B: *EIF1AY* probe ILMN_1755537; C: *EIF1AY* probe ILMN_2228976.

**Note:** Age-expression correlations were calculated using linear regressions, in which inverse normal transformed expression values were regressed on age, with adjustment for smoking status, alcohol drinking status and BMI.
